# Preoperative biomarkers related to inflammation may identify high-risk anastomoses in colorectal cancer surgery: explorative study

**DOI:** 10.1093/bjsopen/zrac072

**Published:** 2022-06-02

**Authors:** Klas Holmgren, Pär Jonsson, Christina Lundin, Peter Matthiessen, Jörgen Rutegård, Malin Sund, Martin Rutegård

**Affiliations:** 1 Department of Surgical and Perioperative Sciences, Surgery, Umeå University, Umeå, Sweden; 2 Department of Chemistry, Umeå University, Umeå, Sweden; 3 Department of Surgery, Faculty of Medicine and Health, School of Health and Medical Sciences, Örebro University, Örebro, Sweden; 4 Department of Surgery, University of Helsinki and Helsinki University Hospital, Helsinki, Finland; 5 Wallenberg Centre for Molecular Medicine, Umeå University, Umeå, Sweden

## Abstract

**Background:**

Colorectal anastomotic leakage can be considered a process of failed wound healing, for which related biomarkers might be a promising research area to decrease leak rates.

**Methods:**

Patients who had elective surgery with a primary anastomosis for non-metastatic colorectal cancer, at two university hospitals between 1 January 2010 and 31 December 2015 were included. Patients with an anastomotic leak were identified and matched (1:1) to complication-free controls on the basis of sex, age, tumour stage, tumour location, and operating hospital. Preoperative blood samples were analysed by use of protein panels associated with systemic or enteric inflammation by proteomics, and enzyme-linked immunosorbent assays. Multivariable projection methods were used in the statistical analyses and adjusted for multiple comparisons to reduce false positivity. Rectal cancer tissue samples were evaluated with immunohistochemistry to determine local expression of biomarkers that differed significantly between cases and controls.

**Results:**

Out of 726 patients undergoing resection, 41 patients with anastomotic leakage were matched to 41 controls. Patients with rectal cancer with leakage displayed significantly elevated serum levels of 15 proteins related to inflammation. After controlling for a false discovery rate, levels of C-X-C motif chemokine 6 (CXCL6) and C-C motif chemokine 11 (CCL11) remained significant. In patients with colonic cancer with leakage, levels of high-sensitivity C-reactive protein (hs-CRP) were increased before surgery. Local expression of CXCL6 and CCL11, and their receptors, were similar in rectal tissues between cases and controls.

**Conclusion:**

Patients with anastomotic leakage could have an upregulated inflammatory response before surgery, as expressed by elevated serological levels of CXCL6 and CCL11 for rectal cancer and hs-CRP levels in patients with colonic cancer respectively.

## Teaser

Anastomotic leakage is a major complication after colorectal surgery, presumed to correspond with a process of failed wound healing, involving inflammation. Circulating levels of inflammation-related biomarkers were investigated in preoperative samples from 41 patients with leakage, who had elective treatment with a primary anastomosis for non-disseminated colorectal cancer, matched to 41 complication-free controls. A total of 15 inflammation-related proteins were elevated before surgery in patients with rectal cancer with leakage, of which C-X-C motif chemokine 6 and C-C motif chemokine 11 remained significantly increased after controlling for multiplicity. As a corresponding expression pattern difference did not emerge when tissue adjacent to the anastomosis was evaluated with immunohistochemistry, findings may reflect a systemic rather than a local host response.

While these findings require validation before implementation into surgical practice, they highlight the need for further translational investigations as a promising research area to help decrease leakage rates.

Preoperative inflammation-related serum proteins were evaluated in a case–control study of 41 patients with anastomotic leakage matched 1:1 with 41 complication-free controls. The chemokines C-X-C motif chemokine 6 and C-C motif chemokine 11 were significantly increased before surgery in patients with rectal cancer and leakage, a finding requiring further validation.

## Introduction

In colorectal surgery, anastomotic leakage remains a serious complication associated with considerable morbidity and decreased survival^1^, and has also been reported to increase the risk of local recurrence^2^. Additionally, leakage is associated with elevated rates of permanent stomas in patients undergoing anterior resection for rectal cancer^3^. This issue has a substantial financial impact on healthcare systems worldwide^4^.

While the pathogenesis behind anastomotic dehiscence is not fully understood^5^, it could be considered to result from a failed wound healing process^6^. Consequently, several studies have investigated postoperative markers of inflammation and tissue injury for early leakage detection^7^; however, in patients with colorectal cancer, altered baseline levels of such biomarkers have been reported^8^ and, even after complete tumour removal, factors correlating with invasion and inflammation remain elevated for several weeks^9^. Interestingly, preoperative systemic inflammation has been associated with a higher prevalence of postoperative infections after colorectal resections, out of which 20 per cent constituted anastomotic leaks^10^. Systemic inflammation, and changes in the local intestinal environment, detectable before surgery, could theoretically persist in the postoperative interval and influence the process of anastomotic healing^11^. Evaluating such biomarkers in patients with colorectal cancer could therefore potentially identify high-risk anastomoses, which in turn could facilitate surgical decision-making.

Within the Uppsala-Umeå Comprehensive Cancer Consortium (U-CAN), blood and tissue samples have been prospectively collected and stored in biobanks for all consenting patients undergoing surgery for colorectal cancer since 2010^12^. Using clinical and biological data from the U-CAN cohort, a hypothesized association between abnormal levels of preoperative biomarkers of inflammation and anastomotic leakage was investigated.

## Methods

### Study design

This was an exploratory matched case–control study approved by the regional ethical review board at Umeå University. Access to blood and tissue samples was approved by the Regional Biobank Centre in Northern Sweden and the U-CAN Diagnosis Specific group for colorectal cancer. All patients included had signed a written consent before any collection of biological material.

From 2010 onwards, blood and tissue samples from patients operated for colorectal cancer at Uppsala and Umeå university hospitals have been collected and stored prospectively within the U-CAN biobank. The U-CAN project encompasses hospitals in two healthcare regions with a catchment population of more than 600,000. The coverage of U-CAN is near complete, with high standards in performance according to recent evaluations, and essentially all patients registered (98 per cent) have had blood samples drawn at the time of inclusion, and with tissue specimens available in 83 per cent^12^. Generally, the samples are collected before any therapeutic interventions, and always before surgery (typically the day before). The blood sampling includes, *inter alia*, EDTA plasma and serum, and the time from blood sampling and obtaining frozen aliquots is generally less than 4 h. Fractions are stored in 0.5-ml microvials (Micronic, Lelystad, The Netherlands) and preserved at ­−80°C. Tissue samples, available as both fresh-frozen and paraffin-embedded, are collected from the surgical specimen; samples are taken both from the tumour itself and from healthy tissue at the resection margins. Additional information about the U-CAN biobank and sampling storage has been outlined in a detailed review^12^.

### Registry data

The Swedish Colorectal Cancer Registry was used to identify patients eligible for study inclusion. It is well validated and encompasses patients diagnosed with colorectal cancer in Sweden, with a near-complete degree of coverage^13^. Reporting to the registry comprises patient-related characteristics, surgical details, postoperative course, final pathological assessment, and a 5-year follow-up. The registry’s definition of rectal cancer is any cancer with its lower margin located within 15 cm of the anal verge, as measured by rigid sigmoidoscopy.

### Anastomotic leakage

For this study, leakage was defined *a priori* (before initiation of the study) as a defect of the intestinal wall at the site of the anastomosis, leading to a communication between the intraluminal and extraluminal compartments, in accordance with an international consensus^14^. Its presence was ascertained by review of medical records. While only leaks within 30 days of surgery are reported to the registry, late leaks diagnosed up to 90 days after surgery were considered eligible and included in the present study. In patients with rectal cancer, a rectovaginal fistula or a pelvic abscess (without radiologically proven leakage) was also considered as a leak^14^. A definitive diagnosis was made by the treating clinicians by use of radiological (rectal contrast study, CT, or MRI), endoscopic (rigid or flexible sigmoidoscopy), or clinical investigations (digital palpation, inspection of drain contents, or verified at reoperation).

### Study cohort and matching

Patients registered to have had elective surgery for colorectal cancer at Umeå and Uppsala University Hospitals between 1 January 2010 and 31 December 2015 were reviewed. Those who did not undergo surgery with construction of a primary anastomosis, or had disseminated disease, were deemed ineligible for study inclusion, after which cases with anastomotic leakage were identified. For every such case, potential controls with a complication-free postoperative course (Clavien–Dindo score 0^15^) were searched for, using the same registry. Matching criteria were sex, age (±5 years, at time of surgery), tumour location (colon or rectum), histopathological assessment of tumour stage (pTNM I, II, or III), and operating hospital (Umeå or Uppsala University Hospital). pTNM stage was cross-checked by examination of histopathological reports. Additionally, to account for possible laboratory immune aberrations sustained by transfusion-related immunomodulation, any allogenic blood transfusion within 90 days before surgery was also noted.

After collection of data, matching was completed. For all potential controls matchable to more than one case, prioritization was performed to ensure all cases had a minimum of one control. Additionally, for all cases that had more than one eligible control, relaxation rules were applied using the following criteria: blood sampling corresponding to sampling completeness of the case (while a preoperative sample was an absolute criterion for study inclusion, postoperative samples were also considered in this context, as the study cohort derives from a larger project that includes different research questions), nearest date of surgery relative to the case, surgical approach (open or laparoscopic), and neoadjuvant treatment (no treatment, radiotherapy, or chemoradiotherapy).

### Proteomic and enzyme-linked immunosorbent assay analyses

Serum samples from all study participants were requested from the biobank and sent for proteomic analysis to Olink Proteomics, Uppsala, Sweden. The predefined biomarker panel ‘Olink inflammation’ (*Table S1*) was selected for analysis, for which a detailed description (including interpretation of the data output) is outlined in the Supplementary material. Based on the findings of previous research on early leakage prediction^11^, levels of high-sensitivity C-reactive protein (hs-CRP) and intestinal fatty acid-binding protein (I-FABP) were also evaluated. As these proteins were not included in the Olink inflammation panel, analyses were performed by enzyme-linked immunosorbent assay (ELISA) with the hs-CRP (Cloud-Clone, Texas, USA) and human I-FABP, ELISA kits (Hyocult Biotech, Uden, The Netherlands). Samples were diluted and analysed in duplicate according to the manufacturers’ protocol. A coefficient of variation (CV) below 10 per cent was deemed acceptable, whereas any duplicate with a CV above 10 per cent would require a repeated analysis.

Last, to further corroborate the findings of the main analyses, a *post hoc* sensitivity analysis was performed by target protein ELISA analyses on residual plasma samples from the subset of patients with rectal cancer who had treatment at Umeå University Hospital exclusively. For this, CXCL6/GCP-2 and CCL11/Eotaxin ELISA kits (R&D Systems, Minnesota, USA) were employed and run as single samples, while otherwise identically performed as the main ELISA analyses.

### Immunohistochemistry

Based on the findings of the primary analyses performed on serum, tissues from patients having surgery for rectal cancer were retrieved. The samples were from the resection specimen margin (close to the anastomosis) and were used to compare chemokine and chemokine receptor expression between cases and controls, by use of immunohistochemistry. The following antibodies were used: anti-C-X-C motif chemokine 6 (CXCL6) (Sigma-Aldrich, Saint Louis, Missouri, USA), anti-Eotaxin/C-C motif chemokine 11 (CCL11) (Abcam, Cambridge, Massachusetts, USA), anti-C-X-C motif chemokine receptor 1 (CXCR1) (Abcam), anti-C-X-C motif chemokine receptor 2 (CXCR2) (Abcam), anti-C-C motif receptor 3 (CCR3) (Sigma-Aldrich), and anti-C-C motif receptor 5 (CCR5) (Sigma-Aldrich). The immunohistochemistry procedure is presented in detail in the Supplementary material. Tissue expression was analysed by light microscopy and representative images were captured.

### Statistical analyses

Categorical variables were presented with the number of observations in each group and percentages, whereas continuous variables were displayed with median and interquartile ranges. Additional information on how variables were handled is available in the Supplementary material.

As outlined previously, the main confounding factors were selected *a priori* and controlled for through matching at time of inclusion, while any remaining baseline differences between cases and controls were evaluated with Fisher’s exact test for categorical variables, and the Student’s *t* test for continuous variables.

In the main analysis, a further developed version of orthogonal projections to latent structures (OPLS)^16^, OPLS-effect projections (OPLS-EP)^17^, designed for dependent 1:1 matched samples, was used. The method employs latent variables, and the probability level of each OPLS-EP model was formally assessed by means of the CV-ANOVA diagnostic^18^. Models with a *P* value below 0.05 were considered significant and used to identify potential biomarkers displaying differences between cases and controls. The interpretation of univariate associations of OPLS-EP models corresponds with that of *t* tests of individual variables^19^, whereas *P* values from two-tailed *t* tests were considered statistically significant at a level below 0.05. Also, to control for a plausible false discovery rate, the Benjamini–Hochberg procedure was used^20^, with a threshold level set at 0.10.

Results of ELISA assays, including the *post hoc* sensitivity analyses, were evaluated with a dependent *t* test and the Wilcoxon signed rank sum test. *P* values were reported and supplemented with a mean fold change and a median fold change to display numerical differences between cases and controls. As only two proteins selected *a priori* (hs-CRP and I-FABP) were covered in the main ELISA analyses, and the *post hoc* estimations were derived from multiplicity-controlled analyses, adjustment for multiple testing were omitted.

Both the biomarker panel analysis and the ELISA analyses were followed by subgroup analyses for patients with colon and rectal cancer separately. Also, to control for any inflammatory response to neoadjuvant radiotherapy, all outcomes were re-evaluated with a sensitivity analysis of patients with concordant oncological treatment.

To illustrate significant serum protein level differences noted between the two groups in the biomarker panel analysis, graphs with distributions divided into tertiles were presented and displayed together with receiver operating characteristic (ROC) curves, along with a grouped discriminant analysis.

Calculations of baseline characteristics and univariate associations were performed with the computer software STATA version 15.1 (StataCorp, Houston, Texas, USA). For OPLS-EP statistics, MATLAB release 2017a (The MathWorks, Natick, Massachusetts, USA) was used.

## Results

**Fig. 1:**
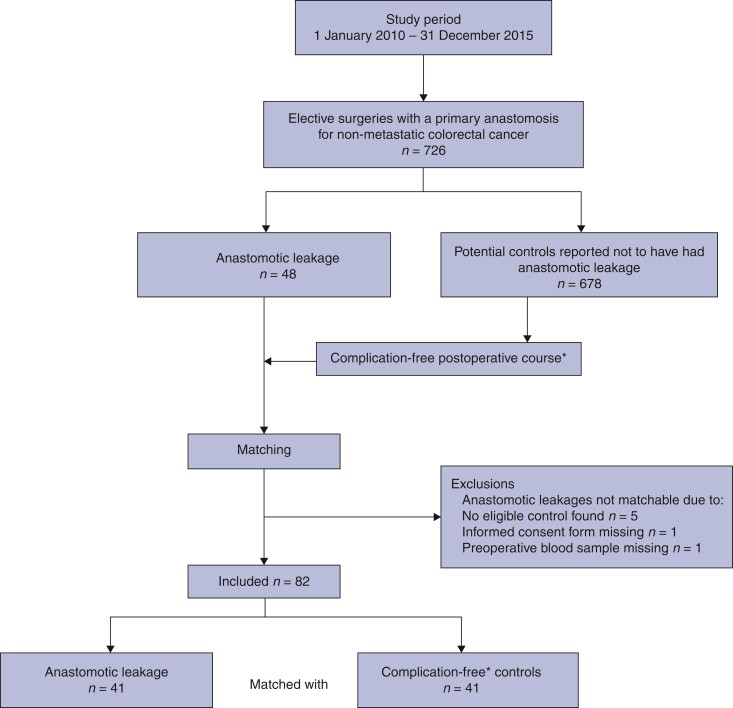
**Study flow chart of patients reported to the Swedish Colorectal Cancer Registry to have undergone elective resection with a primary anastomosis for non-metastatic colorectal cancer at either Uppsala or Umeå university hospitals between 1 January 2010 and 31 December 2015** Anastomotic leakage was determined by review of medical records using an international consensus definition (see main text for further details), while potential controls also underwent chart review. *Complication-free corresponding to a Clavien–Dindo score 0 postoperative course.

During the study interval between 1 January 2010 and 31 December 2015, a total of 726 patients had undergone curative surgery for non-disseminated colorectal cancer with a primary anastomosis at either of the two university hospitals in Uppsala and Umeå, Sweden. As demonstrated in a study flow chart (*Fig. 1*), 48 patients with anastomotic leakage were considered for inclusion, of whom 5 could not be matched as no eligible controls were found; moreover, a missing informed consent and a lost preoperative blood sample resulted in exclusion of 2 more patients. The remaining 41 patients were included and matched 1:1 against 41 controls, amounting to a total of 82 patients in the cohort.

*Table 1* displays patient demographics stratified by anastomotic leakage. While the two groups differed marginally in terms of BMI (*P* = 0.191), cases and controls corresponding to the entire cohort were otherwise comparable. In patients with rectal cancer, groups differed significantly with regard to neoadjuvant treatment (*P* = 0.031).

**Table 1:** Patient characteristics stratified by anastomotic leakage in a cohort of 82 patients who had surgery for non-disseminated colorectal cancer, matched for sex, tumour location, tumour stage, age, and operating hospital

	Complication-free (*n* = 41)	Anastomotic leakage (*n* = 41)	*P*
**Categorical variables**			
**Sex**			
Male	22 (53.7)	22 (53.7)	
Female	19 (46.3)	19 (46.3)	1.00
**BMI (kg/m^2^)**			
<25	19 (46.3)	13 (31.7)	
25–30	17 (41.5)	17 (41.5)	
>30	5 (12.2)	11 (26.8)	0.19
**ASA fitness grade**			
I–II	33 (80.5)	28 (70.0)	
III	8 (19.5)	12 (30.0)	0.31
**Location**			
Colon	24 (58.5)	24 (58.5)	
Rectum	17 (41.5)	17 (41.5)	1.00
Neoadjuvant therapy*			
No neoadjuvant treatment	9 (52.9)	2 (11.8)	
Radiotherapy	4 (23.5)	9 (52.9)	
Chemoradiotherapy	4 (23.5)	6 (35.3)	0.03
**Histopathological tumour stage**			
I	10 (24.4)	10 (24.4)	
II	16 (39.0)	16 (39.0)	
III	15 (36.6)	15 (36.6)	1.00
**Preoperative blood transfusion**†			
No	39 (95.1)	37 (90.2)	
Yes	2 (4.9)	4 (9.8)	0.68
**Surgical approach**‡			
Open	33 (80.5)	32 (78.0)	
Laparoscopic	8 (19.5)	9 (22.0)	0.79
Defunctioning stoma*			
No	3 (17.6)	1 (5.9)	
Yes	14 (82.4)	16 (94.1)	0.34
**Continuous variables**			
**Age (years)**	70 (63–76)	68 (62–76)	0.846
**Intraoperative bleeding (ml)**	100 (50–300)	200 (100–450)	0.362

Values indicate number of observations for categorical variables, median for continuous variables, followed by percentages and interquartile range in parentheses respectively. Fisher’s exact test was used for categorical variables, Student’s *t* test was used for continuous variables. *Patients with rectal cancer only. †Preoperative blood transfusion was considered as any blood transfusion administered within 90 days before surgery. ‡The laparoscopic category included two cases who had conversion to open surgery, evenly distributed between the two outcome groups.

### Biomarker panel analyses by proteomics

In the proteomics panels used in the analysis, approximately 33 per cent of the biomarkers (depending on the subgroup analysed) had more than 10 per cent of samples displaying concentrations outside the limit of detection and were thus excluded (*Table S1*).

When investigating the entire cohort, no differences in measurable serum protein levels were observed between cases and controls. In a subgroup analysis of the 34 patients with rectal cancer, serum levels of 15 proteins associated with inflammation displayed significantly altered baseline levels between cases and controls in the paired analysis (*Table 2*). After adjustment for a plausible false discovery rate, two proteins, CXCL6 and CCL11, remained within the accepted statistical significance threshold. Results did not differ in a sensitivity analysis, where only matched cases and controls who had concordant oncological treatment were considered (CXCL6, *P* = 0.008; CCL11, *P* = 0.026).

**Table 2:** Serum proteins with significantly higher concentrations before surgery in patients who had an anastomotic leak after rectal cancer resection, as referenced to preoperative levels of matched controls with a complication-free postoperative course

Protein	*P* *	FDR†
**C-X-C motif chemokine 6 (CXCL6)**	<0.01	Pass
**Eotaxin (CCL11)**	<0.01	Pass
**Eukaryotic translation initiation factor 4E-binding protein (4E-BP1)**	<0.01	NS
**Natural killer cell receptor 2B4 (CD244)**	<0.01	NS
**C-X-C motif chemokine 1 (CXCL1)**	0.01	NS
**Monocyte chemotactic protein 2 (MCP-2)**	0.01	NS
**C-X-C motif chemokine 11 (CXCL11)**	0.02	NS
**Tumour necrosis factor ligand superfamily member 14 (TNFSF14)**	0.02	NS
**Tumour necrosis factor ligand superfamily member 9 (TNFRSF9)**	0.03	NS
**CD40L receptor (CD40)**	0.03	NS
**Adenosine deaminase (ADA)**	0.03	NS
**C-C motif chemokine 25 (CCL25)**	0.04	NS
**STAM-binding protein (STAMBP)**	0.04	NS
**Caspase-8 (CASP-8)**	0.04	NS
**Leukaemia inhibitory factor receptor (LIF-R)**	0.04	NS

* Using multivariable projections, by means of orthogonal projections to latent structures-effect projections for dependent samples, and univariate associations corresponding to a paired *t* test of individual variables. FDR, false discovery rate. †Benjamini–Hochberg used to adjust for FDR of multiple testing; pass indicates a result remaining significant after controlling for FDR < 0.1, with NS denoting non-significance.

**Fig. 2:**
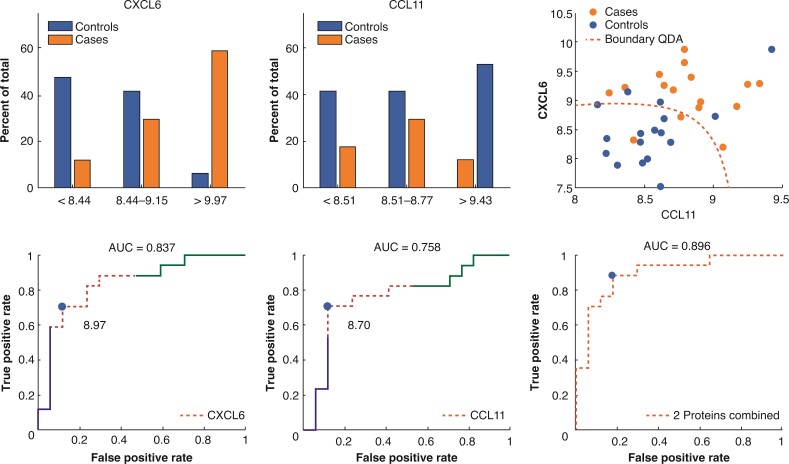
**Preoperative distributions (tertiles) and a grouped discriminant analysis of proteins CXCL6 and CCL11 that were significantly higher in patients with anastomotic leakage after rectal cancer resection (34 cases and controls matched 1:1)** Receiver operating characteristic curves and an estimation of area under the curve (AUC) denoting prediction accuracy. QDA, quadratic discriminant analysis.

*Figure 2* shows the distributions of CXCL6 and CCL11 concentrations between cases and controls, divided into tertiles. Protein levels were evenly distributed between the two groups in the mid tertile, whereas the upper and lower tertiles showed evident discrepancies, with less than 10 per cent of controls present in the upper tertile for both proteins. Accompanying ROC curves illustrate an area under the curve (AUC) of 0.837 for CXCL6 and 0.758 for CCL11, whereas a discriminant analysis of merged data, using both proteins, demonstrated a slightly higher degree of separability between cases and controls with an AUC of 0.896.

### Biomarker analyses by ELISA assays

As demonstrated in *Table 3*, results from ELISA analyses displayed statistically weak, although significant, elevations in preoperative titres of hs-CRP in patients with anastomotic leakage in the entire cohort (mean fold change 3.595, *P* = 0.044; median fold change 2.308, *P* = 0.039) as well as in the colonic cancer subgroup (mean fold change 4.336, *P* = 0.048; median fold change 2.771, *P* = 0.031). In patients with rectal cancer, levels of hs-CRP did not differ between cases and controls. Concentrations of I-FABP were similar between cases and controls. A sensitivity analysis conducted with matched pairs who had concordant neoadjuvant treatment exclusively showed similar results (*Table S2*).

**Table 3:** Comparison of preoperative serum protein levels in 41 patients who had anastomotic leakage after resection for colorectal cancer, with 41 matched controls with a complication-free postoperative course as reference

Protein	Mean fold change	P*	Median fold change	*P*†
**hs-CRP**				
Entire cohort	3.56	0.04	2.30	0.04
Colon	4.34	0.05	2.77	0.03
Rectum	2.55	0.63	1.73	0.47
**I-FABP**				
Entire cohort	2.87	0.73	1.13	0.51
Colon	3.17	0.77	1.72	0.25
Rectum	2.45	0.87	0.82	0.52

* Dependent *t* test for comparison of matched cases and controls. †Wilcoxon signed rank sum test for comparison of matched samples. Entire cohort indicates that all 82 patients were included in the analysis, with ‘colon’ and ‘rectum’ denoting subgroup analyses, with a total of 48 patients in the former group, and 34 patients in the latter group. hs-CRP, high-sensitivity C-reactive protein; I-FABP, intestinal fatty acid-binding protein.

### Tissue expression of chemokines and their receptors

**Fig. 3:**
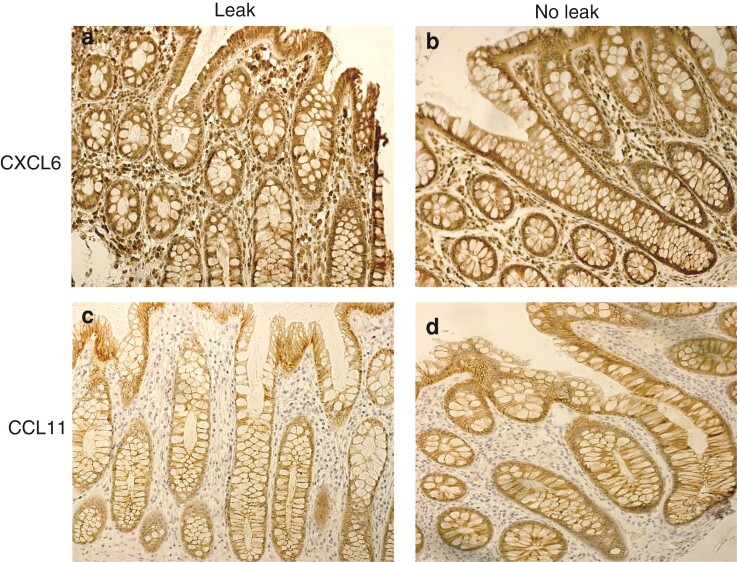
**Representative images of immunohistochemical staining of normal tissue at the resection margin from rectal cancer patients defined as cases (anastomotic leakage) and controls (complication-free postoperative course) using antibodies against CXCL6 and CCL11** **a**,**b** CXCL6 is expressed in the mucosal epithelium and by some stromal cells. **c**,**d** CCL11 is mainly expressed by the epithelium. As illustrated, there were no signs of differences in expression pattern or intensity in patients with anastomotic leakage, and those with a complication-free postoperative course.

As a hypothetical source of the observed differences in circulating CXCL6 and CCL11 levels between rectal cancer cases and controls, immunohistochemical staining of normal tissues from the resection margin displayed no obvious differences between the two groups (*Fig. 3*). Staining was positive for both chemokines and their receptors (*Fig. 3* and *Fig. S1*).

### *Post hoc* sensitivity analyses

To try and further corroborate the main study findings, *post hoc* sensitivity analyses were conducted on residual plasma samples from the 14 rectal cancer resections performed at Umeå University Hospital. Although not reaching formal statistical significance, CXCL6 and CCL11 displayed a corresponding increased trend in patients with anastomotic leakage (*Table 4)*.

**Table 4:** *Post hoc* sensitivity analyses performed on preoperative plasma samples in a matched subset of seven patients who had anastomotic leakage after resection for rectal cancer, with seven complication-free controls as reference

Protein	Mean fold change	*P* *	Median fold change	*P*†
**C-X-C motif chemokine 6 (CXCL6)**	1.49	0.18	1.50	0.16
**Eotaxin (CCL11)**	1.04	0.81	1.18	0.47

* Dependent *t* test for comparison of matched cases and controls. †Wilcoxon signed rank sum test for comparison of matched samples.

## Discussion

In this explorative matched case–control study, patients with rectal cancer with anastomotic leakage had significantly elevated preoperative serum levels of the inflammation-related proteins CXCL6 and CCL11, after correction for multiple comparisons. On the contrary, patients with colonic cancer and anastomotic leakage demonstrated a marginally significant increase in hs-CRP before surgery, whereas titres were similar between cases and controls in the rectal cancer group. No evidence of differences in expression pattern and intensity of CXCL6 or CCL11 and their corresponding protein receptors in rectal cancer as well as healthy tissue could be found by immunohistochemical staining; however, plasma levels of CXCL6 and CCL11 were increased, though not to a statistically significant degree.

There are several limitations to the present study. While the conservative matching criteria are on the one hand considered a study strength and necessary to avoid any baseline differences in serum protein expressions between the pairs, a few potential cases were lost due to a lack of eligible controls. Notwithstanding, a discrepancy remained in the use of neoadjuvant therapy within the rectal cancer group, likely a result from matching based on histopathology rather than clinical tumour stage. As radiotherapy has been associated with leakage^21^ (although this is controversial^22^) and constitutes a plausible driver for an elevated inflammatory state, it can be noted that sensitivity analyses with matching for neoadjuvant therapy nevertheless demonstrated consistent results. Moreover, the explorative nature of the present report with numerous biomarkers analysed entails a risk of false-positive discoveries, although this was controlled for in part by means of the Benjamini–Hochberg procedure. The limited statistical power also raises some concerns, although the sample size is comparable to previous studies on leakage pathophysiology^11,23^. It is important to bear in mind that formal validation in prospective settings is required before any firm conclusions concerning the clinical utility of the derived biomarkers can be drawn. Furthermore, assessment through a single preoperative blood sample incompletely covers the dynamics of inflammation. Ideally samples would be collected at multiple time points, and by taking potential circadian variations into consideration.

While only two proteins, CXCL6 and CCL11, remained significant after controlling for multiple testing, all 15 inflammation-related biomarkers in the primary Olink panel analysis displayed elevated serum concentrations before surgery in patients with rectal cancer and anastomotic leakage; however, preoperative levels of hs-CRP were statistically significantly elevated only in patients with colonic cancer and subsequent leakage, although this finding could be considered weak, as formal statistical significance was only just reached, and the corresponding fold changes were similar in patients with rectal cancer. These conflicting results might therefore be a consequence of a small sample size. In addition, as the ELISA analyses were selected beforehand, adjusting for multiple testing was omitted, which might be another plausible source of this disagreement. Also, while preoperative systemic inflammation correlates with postoperative complications in general^10^, previous reports focusing on anastomotic leakage have failed to demonstrate a corresponding mechanism for CRP, both in terms of CRP in itself^24^, but also for interleukin-6, which stimulates production of CRP^11^. Altogether, the absence of an increase in hs-CRP levels in patients with rectal cancer may suggest that preoperative systemic inflammation was not a major driver of anastomotic leakage in these patients, whereas a risk of type I errors needs to be taken into account for the contrasting results regarding patients with colonic cancer. On the contrary, levels of CXCL6 and CCL11 showed strong correlations with anastomotic leakage in patients with rectal cancer, further supported by the sensitivity analysis.

It is important to bear in mind that local inflammation constitutes a physiological phase of normal wound healing; however, dysregulated inflammation (excessively activated or prolonged) is well known to impair wound healing^25^. For instance, in wounds where microbial clearance is incomplete, sustained inflammation increases the risk of dehiscence, which has largely been attributed to increased levels of matrix metalloproteases (MMPs) that degrade extracellular matrix^25^. Interestingly, CXCL6 is known to stimulate and secrete proteases, such as MMP-9 from granulocytes^26^, and may indicate that such protease activity was higher in those with anastomotic failure. Moreover, CXCL6 has also been ascribed with strong antibacterial properties^27^. Hypothetically, high levels of CXCL6 in patients who had a leak may reflect an unfavourable intestinal microbiome, due to bacterial dysregulation of inflammatory systems, as previously substantiated by Shogan and colleagues^28^, and a recent review^29^. Altogether, the proteolytic activity of MMP-9 constitutes a conceivable driver of anastomotic breakdown, wherein bacterial agents with a high level of such collagenase activity seem to play an important role in the leakage pathogenesis^28^; this has also been conceptualized in the healing of experimental anastomoses by inhibiting MMP activity through antibiotic treatment^30^.

CCL11 is a powerful eosinophil attractant that has been suggested to play an important role in several chronic inflammatory conditions, and especially in diseases affecting the gastrointestinal tract^31^. While CCL11 concentrations are elevated in patients with inflammatory bowel disease, serum levels are significantly higher in those with active disease^32^, and CCL11 is strongly suggested to play a pivotal role in development of mucosal inflammation in these conditions^33^. Hypothetically, higher CCL11 expressions in patients with anastomotic failure in the present cohort may therefore suggest that mucosal inflammation was increased, providing a luminal environment with unfavourable conditions, risking bowel wall breakdown and intestinal fistulation; however, no such pattern emerged in an attempt to verify whether local differences in the tissue expression pattern and intensity of both CXCL6 and CCL11 and their cellular receptors would explain the observed differences in circulating protein levels. Speculatively, this may suggest that healthy and tumour tissue itself play a lesser role in the upregulation of these circulating proteins, while other patient and systemic factors may require further study to better understand the mechanisms involved.

Furthermore, a previous study found that intestinal fatty acid-binding protein (I-FABP), a marker of enterocyte damage, was elevated before surgery in patients with rectal cancer who later had anastomotic leakage^11^. As intestinal barrier dysfunction denoted by I-FABP correlates with a shift in gut microbiota^34^, it is of interest that a corresponding loss of cytoprotectives in patients with I-FABP elevation and leakage has been reported^28^. While uncorroborated by the present study findings in terms of I-FABP, CXCL6, is strongly associated with mucosal infections^27^, and was clearly elevated in patients with leakage. This finding might further substantiate the concept that gut inflammation and intestinal microbiome play an important role in anastomotic healing. Moreover, while prognostic information concerning colorectal leaks has been derived from expression of interleukins and tumour necrosis factor during the early postoperative interval, a similar pattern did not emerge based on the preoperative samples studied herein^35^.

While the exact interplay between gut microbiota, inflammatory response, and anastomotic leakage stretches beyond the scope of the present study, the findings herein of biomarkers for mucosal inflammation and bacterial activity may help shed further light on why some anastomoses fail to heal. The results should be interpreted with great caution with respect to the study limitations; however, anastomotic leakage in surgery for rectal cancer remains a challenge, where even provisional findings warrant further study and formal validation.

## Supplementary Material

zrac072_Supplementary_Data

## Data Availability

Upon reasonable request, data and methodology, including STATA and MATLAB software code, can be shared. This also applies to the registry-based data and results from analyses performed on biological material, while access to such data might be subject to external review by the Swedish Colorectal Cancer Registry steering committee, and biobank custodian respectively.

## References

[1] McArdle CS , McMillan DC, Hole DJ. Impact of anastomotic leakage on long-term survival of patients undergoing curative resection for colorectal cancer. Br J Surg 2005;92:1150–1154 16035134 10.1002/bjs.5054

[2] Lu ZR , Rajendran N, Lynch AC, Heriot AG, Warrier SK. Anastomotic leaks after restorative resections for rectal cancer compromise cancer outcomes and survival. Dis Colon Rectum 2016;59:236–244 26855399 10.1097/DCR.0000000000000554

[3] Holmgren K , Kverneng Hultberg D, Haapamäki MM, Matthiessen P, Rutegård J, Rutegård M. High stoma prevalence and stoma reversal complications following anterior resection for rectal cancer: a population-based multicentre study. Colorectal Dis 2017;19:1067–1075 28612478 10.1111/codi.13771

[4] La Regina D , Di Giuseppe M, Lucchelli M, Saporito A, Boni L, Efthymiou C et al Financial impact of anastomotic leakage in colorectal surgery. J Gastrointest Surg 2019;23:580–586 30215201 10.1007/s11605-018-3954-z

[5] Shogan BD , Carlisle EM, Alverdy JC, Umanskiy K. Do we really know why colorectal anastomoses leak? J Gastrointest Surg 2013;17:1698–1707 23690209 10.1007/s11605-013-2227-0

[6] Thompson SK , Chang EY, Jobe BA. Clinical review: healing in gastrointestinal anastomoses, Part I. Microsurgery 2006;26:131–136 16518804 10.1002/micr.20197

[7] Hirst NA , Tiernan JP, Millner PA, Jayne DG. Systematic review of methods to predict and detect anastomotic leakage in colorectal surgery. Colorectal Disease 2014;16:95–109 23992097 10.1111/codi.12411

[8] Kantola T , Vayrynen JP, Klintrup K, Makela J, Karppinen SM, Pihlajaniemi T et al Serum endostatin levels are elevated in colorectal cancer and correlate with invasion and systemic inflammatory markers. Br J Cancer 2014;111:1605–1613 25137019 10.1038/bjc.2014.456 PMC4200096

[9] Kumara HM S , Tohme ST, Yan X, Nasar A, Senagore AJ, Kalady MF et al Plasma levels of angiostatin and endostatin remain unchanged for the first 3 weeks after colorectal cancer surgery. Surg Endosc 2011;25:1939–1944 21181203 10.1007/s00464-010-1491-2

[10] Moyes LH , Leitch EF, McKee RF, Anderson JH, Horgan PG, McMillan DC. Preoperative systemic inflammation predicts postoperative infectious complications in patients undergoing curative resection for colorectal cancer. Br J Cancer 2009;100:1236–1239 19319134 10.1038/sj.bjc.6604997 PMC2676538

[11] Reisinger KW , Poeze M, Hulsewe KWE, van Acker BA, van Bijnen AA, Hoofwijk AGM et al Accurate prediction of anastomotic leakage after colorectal surgery using plasma markers for intestinal damage and inflammation. J Am Coll Surg 2014;219:744–751 25241234 10.1016/j.jamcollsurg.2014.06.011

[12] Glimelius B , Melin B, Enblad G, Alafuzoff I, Beskow A, Ahlström H et al U-CAN: a prospective longitudinal collection of biomaterials and clinical information from adult cancer patients in Sweden. Acta Oncol 2018;57:187–194 28631533 10.1080/0284186X.2017.1337926

[13] Moberger P , Sköldberg F, Birgisson H. Evaluation of the Swedish Colorectal Cancer Registry: an overview of completeness, timeliness, comparability and validity. Acta Oncol 2018;57:1611–1621 30477372 10.1080/0284186X.2018.1529425

[14] Rahbari NN , Weitz J, Hohenberger W, Heald RJ, Moran B, Ulrich A et al Definition and grading of anastomotic leakage following anterior resection of the rectum: a proposal by the International Study Group of Rectal Cancer. Surgery 2010;147:339–351 20004450 10.1016/j.surg.2009.10.012

[15] Dindo D , Demartines N, Clavien PA. Classification of surgical complications: a new proposal with evaluation in a cohort of 6336 patients and results of a survey. Ann Surg 2004;240:205–213 15273542 10.1097/01.sla.0000133083.54934.ae PMC1360123

[16] Trygg J , Wold S. Orthogonal projections to latent structures (O-PLS). J Chemom 2002;16:119–128

[17] Jonsson P , Wuolikainen A, Thysell E, Chorell E, Stattin P, Wikstrom P et al Constrained randomization and multivariate effect projections improve information extraction and biomarker pattern discovery in metabolomics studies involving dependent samples. Metabolomics 2015;11:1667–1678 26491420 10.1007/s11306-015-0818-3 PMC4605978

[18] Eriksson L , Trygg J, Wold S. CV-ANOVA for significance testing of PLS and OPLS (R) models. J Chemom 2008;22:594–600

[19] Jonsson P , Björkblom B, Chorell E, Olsson T, Antti H. Statistical loadings and latent significance simplify and improve interpretation of multivariate projection models. BioRxiv 2018; DOI: 10.1101/350975 [Epub ahead of print]

[20] Benjamini Y , Hochberg Y. Controlling the false discovery rate - a practical and powerful approach to multiple testing. J R Stat Soc Series B Stat Methodol 1995;57:289–300

[21] Qin Q , Ma T, Deng Y, Zheng J, Zhou Z, Wang H et al Impact of preoperative radiotherapy on anastomotic leakage and stenosis after rectal cancer resection: post hoc analysis of a randomized controlled trial. Dis Colon Rectum 2016;59:934–942 27602924 10.1097/DCR.0000000000000665

[22] Hu MH , Huang RK, Zhao RS, Yang KL, Wang H. Does neoadjuvant therapy increase the incidence of anastomotic leakage after anterior resection for mid and low rectal cancer? A systematic review and meta-analysis. Colorectal Dis 2017;19:16–26 27321374 10.1111/codi.13424

[23] Alonso S , Pascual M, Salvans S, Mayol X, Mojal S, Gil MJ et al Postoperative intra-abdominal infection and colorectal cancer recurrence: a prospective matched cohort study of inflammatory and angiogenic responses as mechanisms involved in this association. Eur J Surg Oncol 2015;41:208–214 25468742 10.1016/j.ejso.2014.10.052

[24] Almeida AB , Faria G, Moreira H, Pinto-de-Sousa J, Correia-da-Silva P, Maia JC. Elevated serum C-reactive protein as a predictive factor for anastomotic leakage in colorectal surgery. Int J Surg 2012;10:87–91 22222182 10.1016/j.ijsu.2011.12.006

[25] Guo S , Dipietro LA. Factors affecting wound healing. J Dent Res 2010;89:219–229 20139336 10.1177/0022034509359125 PMC2903966

[26] Emmanouil G , Ayiomamitis G, Zizi-Sermpetzoglou A, Tzardi M, Moursellas A, Voumvouraki A et al Angiodrastic chemokines in colorectal cancer: clinicopathological correlations. Anal Cell Pathol (Amst) 2018; 2018:1616973 29850390 10.1155/2018/1616973 PMC5926520

[27] Linge HM , Collin M, Nordenfelt P, Mörgelin M, Malmsten M, Egesten A. The human CXC chemokine granulocyte chemotactic protein 2 (GCP-2)/CXCL6 possesses membrane-disrupting properties and is antibacterial. Antimicrob Agents Chemother 2008;52:2599–2607 18443119 10.1128/AAC.00028-08 PMC2443903

[28] Shogan BD , Belogortseva N, Luong PM, Zaborin A, Lax S, Bethel C et al Collagen degradation and MMP9 activation by Enterococcus faecalis contribute to intestinal anastomotic leak. Sci Transl Med 2015;7:286ra68 10.1126/scitranslmed.3010658 PMC5027898 25947163

[29] Gaines S , Shao C, Hyman N, Alverdy JC. Gut microbiome influences on anastomotic leak and recurrence rates following colorectal cancer surgery. Br J Surg 2018;105:e131–e141 29341151 10.1002/bjs.10760 PMC5903685

[30] Siemonsma MA , de Hingh IH, de Man BM, Lomme RM, Verhofstad AA, Hendriks T. Doxycycline improves wound strength after intestinal anastomosis in the rat. Surgery 2003;133:268–276 12660638 10.1067/msy.2003.27

[31] Williams TJ . Eotaxin-1 (CCL11). Front Immunol 2015;6:84 25759694 10.3389/fimmu.2015.00084 PMC4338813

[32] Chen W , Paulus B, Shu D, Wilson CV. Increased serum levels of eotaxin in patients with inflammatory bowel disease. Scand J Gastroenterol 2001;36:515–520 11346206 10.1080/003655201750153377

[33] Adar T , Shteingart S, Ben-Ya'acov A, Shitrit AB, Livovsky DM, Shmorak S et al The importance of intestinal eotaxin-1 in inflammatory bowel disease: new insights and possible therapeutic implications. Dig Dis Sci 2016;61:1915–1924 26874691 10.1007/s10620-016-4047-z

[34] Lau E , Marques C, Pestana D, Santoalha M, Carvalho D, Freitas P et al The role of I-FABP as a biomarker of intestinal barrier dysfunction driven by gut microbiota changes in obesity. Nutr Metab (Lond) 2016;13:31 27134637 10.1186/s12986-016-0089-7 PMC4851788

[35] Sparreboom CL , Wu Z, Dereci A, Boersema GS, Menon AG, Ji J et al Cytokines as early markers of colorectal anastomotic leakage: a systematic review and meta-analysis. Gastroenterol Res Pract 2016;2016:s3786418 10.1155/2016/3786418 PMC4804081 27051416

