# Supplementary material for: Preoperative biomarkers related to inflammation may identify high-risk anastomoses in colorectal cancer surgery: explorative study
Source: BJS Open. 2022 Jun 2;6(3):zrac072. doi: 10.1093/bjsopen/zrac072 (PMC9161645; doi:10.1093/bjsopen/zrac072)
Supplement: zrac072_Supplementary_Data [file zrac072_supplementary_data.docx]

### Supplementary material

### Olink panel analyses

The Olink analyses are run through adding 92-protein-specific pairs of antibodies labelled with unique complementary oligonucleotides (PEA probes) to 1 µl of each serum sample. The antibody pairs target a specific protein and, once attached to their target, the unique PEA probes nearby allow for hybridization, which in turn generates a DNA template that can act as a protein marker. Templates are thereafter extended by means of DNA polymerase and pre-amplified by quantitative polymerase chain reaction (qPCR) using universal primers. Excess primers are cleared before quantification by a microfluidic chip (96.96 Dynamic array IFC, Fluidigm Biomark), which is run in a qPCR platform (BioMark HD System). For more information, detailed descriptions are readily available through the company website^1^. All analyses were performed blinded to the study endpoint.

The biomarker panel analysis generates output in the form of Normalized Protein eXpression (NPX), which is an arbitrary unit on the log2 scale. Consequently, measurements are only applicable for relative quantification for each protein individually between samples, and noncomparable in terms of absolute numbers or between analyses performed at different time points. With results derived on a log2 scale, each one-unit increase in NPX corresponds to a doubling in protein concentration. Furthermore, proteins with more than 10% of NPX results with concentrations outside the limit of detection (LOD) were excluded (*Table S1,* supporting information).

### Immunohistochemistry

Paraffin-embedded tissue specimens from rectal cancer patients who underwent surgical resection were retrieved from the U-CAN biobank. Five μm sections were cut and used for immunohistochemical staining using the following antibodies and dilutions; anti-C-X-C motif chemokine 6 (CXCL6, 1:200) (Sigma-Aldrich, Saint Louis, MO, USA), anti-Eotaxin/C-C motif chemokine 11 (CCL11; 1:50) (Abcam, Cambridge, MA, USA), anti-C-X-C motif chemokine receptor 1 (CXCR1, 1:100) (Abcam), anti-C-X-C motif chemokine receptor 2 (CXCR2, 1:100) (Abcam), anti-C-C motif receptor 3 (CCR3, 1:50) (Sigma-Aldrich) and anti-C-C motif receptor 5 (CCR5, 1:300) (Sigma-Aldrich). Tissues were deparaffinized and rehydrated, followed by blocking of endogenous peroxidase activity in 3% H_2_O_2_ solution. After rinsing, antigen retrieval was performed using EDTA for all antibodies except anti-CXCR1 where done by citrate buffer. Tissue sections were then further blocked, followed by incubation with the primary antibodies (1 hour at room temperature (RT)). Subsequently, secondary antibodies linked to horseradish peroxidase were added for 30 min at room temperature, followed by rinsing and adding diaminobenzidine tetrahydrochloride as a chromogen, and finally counterstaining using haematoxylin.

### Statistical analyses

BMI was classified using the conventional taxonomy (<25 kg/m^2^: normal weight; 25–30 kg/m^2^: overweight; >30 kg/m^2^: obesity), while neoadjuvant treatment was divided into three different categories (no neoadjuvant treatment, radiotherapy or chemoradiotherapy). Histopathological tumour stage (pTNM) was also treated as a categorical variable, retaining all three classes (I, II and III) separated. Sex (male or female), tumour location (colon or rectum), preoperative blood transfusion (no or yes) and defunctioning stoma (no or yes) were managed as binary variables. Due to small numbers and similar relations to the main outcome, ASA classes I and II were merged into a reference category, while ASA III was treated as a separate category owing to a possible association to anastomotic leakage^2, 3^. The variables age and intraoperative bleeding were kept as continuous and presented with interquartile ranges.

### Supporting references

1. Olink Proteomics. <https://www.olink.com/> [accessed 28 October 2021].

2. Gessler B, Bock D, Pommergaard HC, Burcharth J, Rosenberg J, Angenete E. Risk factors for anastomotic dehiscence in colon cancer surgery--a population-based registry study. *Int J Colorectal Dis* 2016;**31**(4): 895-902.

3. Jannasch O, Klinge T, Otto R, Chiapponi C, Udelnow A, Lippert H, Bruns CJ, Mroczkowski P. Risk factors, short and long term outcome of anastomotic leaks in rectal cancer. *Oncotarget* 2015;**6**(34): 36884-36893.

| **Table S1** Proteins (*n* = 92) included in the Olink Proteomics inflammation panel | | | |  |
| --- | --- | --- | --- | --- |
| **Protein** (Abbreviation) | *Entire cohort* | *Colon* | *Rectum* | |
| **IL8** |  |  |  |  |
| **VEGFA** |  |  |  |  |
| **MCP-3** |  |  | *LOD* |  |
| **GDNF** | *LOD* | *LOD* | *LOD* |  |
| **CDCP1** |  |  |  |  |
| **CD244** |  |  |  |  |
| **IL7** |  |  |  |  |
| **OPG** |  |  |  |  |
| **LAP TGF-beta-1** |  |  |  |  |
| **uPA** |  |  |  |  |
| **IL6** | *LOD* | *LOD* | *LOD* |  |
| **IL-17C** | *LOD* | *LOD* | *LOD* |  |
| **MCP-1** |  |  |  |  |
| **IL-17A** | *LOD* | *LOD* | *LOD* |  |
| **CXCL11** |  |  |  |  |
| **AXIN1** | *LOD* | *LOD* | *LOD* |  |
| **TRAIL** |  |  |  |  |
| **IL-20RA** | *LOD* | *LOD* | *LOD* |  |
| **CXCL9** |  |  |  |  |
| **CST5** |  |  |  |  |
| **IL-2RB** | *LOD* | *LOD* | *LOD* |  |
| **IL-1 alpha** | *LOD* | *LOD* | *LOD* |  |
| **OSM** |  |  |  |  |
| **IL2** | *LOD* | *LOD* | *LOD* |  |
| **CXCL1** |  |  |  |  |
| **TSLP** | *LOD* | *LOD* | *LOD* |  |
| **CCL4** |  |  |  |  |
| **CD6** |  |  |  |  |
| **SCF** |  |  |  |  |
| **IL18** |  |  |  |  |
| **SLAMF1** | *LOD* | *LOD* | *LOD* |  |
| **TGF-alpha** |  |  |  |  |
| **MCP-4** |  |  |  |  |
| **CCL11** |  |  |  |  |
| **TNFSF14** |  |  |  |  |
| **FGF-23** | *LOD* | *LOD* | *LOD* |  |
| **IL-10RA** | *LOD* | *LOD* | *LOD* |  |
| **FGF-5** | *LOD* | *LOD* | *LOD* |  |
| **MMP-1** |  |  |  |  |
| **LIF-R** |  |  |  |  |
| **FGF-21** |  |  |  |  |
| **CCL19** |  |  |  |  |
| **IL-15RA** | *LOD* | *LOD* | *LOD* |  |
| **IL-10RB** |  |  |  |  |
| **IL-22 RA1** | *LOD* | *LOD* | *LOD* |  |
| **IL-18R1** |  |  |  |  |
| **PD-L1** |  |  |  |  |
| **Beta-NGF** |  |  |  |  |
| **CXCL5** |  |  |  |  |
| **TRANCE** |  |  |  |  |
| **HGF** |  |  |  |  |
| **IL-12B** |  |  |  |  |
| **IL-24** | *LOD* | *LOD* | *LOD* |  |
| **IL13** | *LOD* | *LOD* | *LOD* |  |
| **ARTN** | *LOD* | *LOD* | *LOD* |  |
| **MMP-10** |  |  |  |  |
| **IL10** |  |  |  |  |
| **TNF** | *LOD* | *LOD* | *LOD* |  |
| **CCL23** |  |  |  |  |
| **CD5** |  |  |  |  |
| **CCL3** |  |  |  |  |
| **Flt3L** |  |  |  |  |
| **CXCL6** |  |  |  |  |
| **CXCL10** |  |  |  |  |
| **4E-BP1** |  |  |  |  |
| **IL-20** | *LOD* | *LOD* | *LOD* |  |
| **SIRT2** | *LOD* | *LOD* | *LOD* |  |
| **CCL28** |  |  |  |  |
| **EN-RAGE** |  |  |  |  |
| **CD40** |  |  |  |  |
| **IL33** | *LOD* | *LOD* | *LOD* |  |
| **IFN-gamma** | *LOD* | *LOD* | *LOD* |  |
| **FGF-19** |  |  |  |  |
| **IL4** | *LOD* | *LOD* | *LOD* |  |
| **LIF** | *LOD* | *LOD* | *LOD* |  |
| **NRTN** | *LOD* | *LOD* | *LOD* |  |
| **MCP-2** |  |  |  |  |
| **CASP-8** |  |  |  |  |
| **CCL25** |  |  |  |  |
| **CX3CL1** |  |  |  |  |
| **TNFRSF9** |  |  |  |  |
| **NT-3** | *LOD* | *LOD* | *LOD* |  |
| **TWEAK** |  |  |  |  |
| **CCL20** |  |  |  |  |
| **ST1A1** |  |  |  |  |
| **STAMPB** |  |  |  |  |
| **IL5** | *LOD* | *LOD* | *LOD* |  |
| **ADA** |  |  |  |  |
| **TNFB** |  |  |  |  |
| **CSF-1** |  |  |  |  |
| **DNER** |  |  |  |  |
| **CD8A** | *LOD* | *LOD* | *LOD* |  |
| **Total** | *n = 30* | *n = 30* | *n = 31* |  |
| Entire cohort indicates that all 82 patients were considered, with Colon and Rectum denoting subgroup analyses, with a total of 48 patients in the former group, and 34 patients in the latter group; Limit of Detection (LOD), more than 10% of samples with concentrations outside the limit of detection. | | | |  |

| **Table S2** Comparison of preoperative serum protein levels in patients who had anastomotic leakage after resection for colorectal cancer, with matched controls with a complication-free postoperative course as reference. Results are derived from sensitivity analyses where only patients with concordant neoadjuvant therapy were included | | | | |
| --- | --- | --- | --- | --- |
| **Protein** | *Mean fold-change* | *P** | *Median fold-change* | *P†* |
| **hs-CRP** |  |  |  |  |
| Entire cohort | 3.63 | 0.05 | 2.30 | 0.06 |
| Colon | 4.34 | 0.05 | 2.77 | 0.03 |
| Rectum | 2.52 | 0.75 | 1.17 | 0.64 |
| **I-FABP** |  |  |  |  |
| Entire cohort | 2.96 | 0.60 | 1.15 | 0.34 |
| Colon | 3.17 | 0.77 | 1.72 | 0.25 |
| Rectum | 2.65 | 0.62 | 0.95 | 0.77 |
| *Dependent t-test for comparison of matched cases and controls; †Wilcoxon signed rank sum test for comparison of matched samples; hs-CRP, high-sensitivity C-reactive protein; Entire cohort indicates that all 64 patients were included in the sensitivity analysis, with Colon and Rectum denoting subgroup analyses, with a total of 44 patients in the former group, and 20 patients in the latter group; I-FABP, Intestinal fatty acid-binding protein. | | | | |

**Fig. S1** Representative images of immunohistochemical staining of normal tissue at the resection margin from rectal cancer patients defined as cases (leak) and controls (no leak) using antibodies against CXCR1, CXCR1, CCR3 and CCR5. CXCR1 is mostly expressed in the basal layers of the mucosal epithelium (A and B) whereas the other chemokine receptors are expressed by epithelial as well as stromal cells (C-H). There were no differences in expression pattern or intensity in patients with leakage, and those with a complication-free postoperative course.
